# Impact of early goal-directed mobilization on ICU-acquired weakness: a meta-analysis

**DOI:** 10.3389/fneur.2026.1922493

**Published:** 2026-09-30

**Authors:** Jiayi Zhou, Lifei Dai, Xiaoli Liu, Junlian Xiang

**Affiliations:** 1 School of Basic Medical Sciences & School of Nursing, Chengdu University, Chengdu, Sichuan, China; 2 Department of Interventional Radiology, Deyang People’s Hospital, Deyang, Sichuan, China; 3 Department of Urology, Deyang People’s Hospital, Deyang, Sichuan, China

**Keywords:** early goal-directed mobilization, early rehabilitation, ICU-acquired weakness syndrome, intensive care unit, mechanical ventilation, meta-analysis

## Abstract

**Background:**

ICU-acquired weakness (ICU-AW) significantly impairs muscle strength and physical function in critically ill patients. Early mobilization interventions have been increasingly adopted, yet the specific effects of a structured EGDM program on ICU-AW and functional outcomes have not been systematically quantified. This meta-analysis aimed to evaluate the effects of EGDM on ICU-AW, muscle strength, and functional outcomes.

**Methods:**

PubMed, Web of Science, Embase, Cochrane Library, China National Knowledge Infrastructure (CNKI), Wanfang Data, and Viper Information Products (VIP) databases were searched for randomized controlled trials (RCTs) up to November 30, 2025. Two reviewers independently screened studies, extracted data, and assessed risk of bias using RoB 2.0. RevMan 5.4 was used for meta-analysis. Subgroup analysis was prespecified by allocation concealment.

**Results:**

Nine RCTs (907 patients) were included. EGDM reduced ICU-AW [*RR* = 0.57, 95%*CI* (0.33 ~ 0.97), *p =* 0.04 for the overall effect*; I^2^* = 77%*, p =* 0.002 for heterogeneity], but sensitivity analysis of high-quality trials showed no significant effect [*RR* = 0.84, 95%*CI* (0.48 ~ 1.46), *p* = 0.54 for the overall effect]. EGDM improved muscle strength [*MD* = 4.94, 95%*CI* (3.39 ~ 6.50), *I^2^* = 59%, *p* = 0.02 for heterogeneity], shortened the duration **of** mechanical ventilation [*MD* = −81.95 h, 95%*CI* (−115.45 ~ −48.46), *p* < 0.00001 for the overall effect; *I^2^* = 85%, *p* < 0.00001 for heterogeneity], and reduced ICU stay[*MD* = -4.55 days, 95%*CI* (−6.22 ~ −2.89), *p* < 0.00001 for the overall effect; *I^2^* = 74%, *p* = 0.0004 for heterogeneity], both with substantial heterogeneity. Although the primary Barthel Index analysis was not statistically significant, sensitivity analysis suggested improvement in patients with moderate baseline function [*MD* = 8.27, 95%*CI* (5.66 ~ 10.87), *p* < 0.00001 for the overall effect].

**Conclusion:**

EGDM may improve muscle strength and physical function in ICU patients. The available evidence does not confirm a reduction in ICU-AW. The effects on ventilation duration and ICU stay varied substantially across studies, likely influenced by sedation protocols, weaning practices, and other context-specific factors. Further rigorous trials with detailed reporting of interventions and contextual factors are needed to guide precise clinical implementation.

**Systematic review registration:**

https://www.crd.york.ac.uk/PROSPERO/view/CRD420261280603, CRD420261280603.

## 1 Introduction

ICU-acquired weakness (ICU-AW) is a syndrome of unexplained weakness in critically ill patients, characterized by difficulty in weaning from mechanical ventilation, paresis or quadriplegia, reduced deep tendon reflexes, and muscle atrophy, with a pathophysiological basis of muscle system dysfunction leading to weakness. Its incidence ranges from 24 to 74% (1). This complication significantly increases mortality, prolongs mechanical ventilation and ICU length of stay, and leads to long-term physical disability and reduced quality of life, making it a critical factor affecting long-term outcomes of critically ill patients (2–4).

Early rehabilitation activities have been shown to be a core strategy for preventing and improving ICU-AW (5, 6). The 2018 clinical practice guidelines for ICU patients recommend early rehabilitation activities for critically ill patients (7), which may shorten the duration of mechanical ventilation, reduce ICU length of stay, and promote functional recovery (8). Among these, goal-directed, structured early rehabilitation programs have gained increasing attention (9).

Early Goal-Directed Mobilization (EGDM), implemented through multidisciplinary collaboration, uses the ICU Mobility Scale (IMS) (10, 11) to assess patients’ maximum activity capacity, formulate daily individualized early rehabilitation activity goals, and implement a structured activity guidance plan to motivate patients to achieve these goals, thereby improving outcomes. Throughout implementation, continuous monitoring ensures physiological stability (12). Unlike the broader concept of early mobilization, which encompasses a range of activities and may vary in its timing, intensity, and progression across studies, EGDM emphasizes a goal-directed process in which systematic assessment is linked to individualized mobility goals and the subsequent progression of activity. Thus, the goal-directed nature of EGDM lies not simply in initiating mobilization early, but in using patient-specific assessment and goals to guide the selection and progression of mobilization.

However, in Chinese ICU practice, the implementation rate of early rehabilitation activities for mechanically ventilated patients remains low, and there is a lack of systematic, operational protocols (13). Although previous studies have investigated early mobilization and goal-directed approaches, the specific components and implementation characteristics of EGDM have not been uniformly described (14). In the present study, EGDM was operationally defined as an intervention incorporating three essential elements: individualized goal setting, structured assessment, and multidisciplinary team involvement. Individualized goal setting establishes patient-specific mobilization targets; structured assessment provides a basis for determining the appropriate level of activity; and multidisciplinary involvement supports the planning and implementation of the mobilization program. Together, these elements characterize the goal-directed and progressive nature of EGDM. Although the specific content, timing, and progression of mobilization may vary according to patients’ clinical status and individual study protocols, the three predefined components provide a consistent framework for identifying the goal-directed nature of the intervention across studies.

To provide a clear and reproducible basis for identifying the intervention of interest, the present review applied this operational definition when selecting eligible studies. This study aims to adopt a strict operational definition of EGDM (i.e., EGDM incorporating goal-setting, structured assessment, and multidisciplinary team as three essential elements) (15) and to conduct a meta-analysis to comprehensively evaluate the effects of EGDM on the incidence of ICU-AW and muscle strength. The findings are intended to provide more robust evidence to inform clinical practice.

## 2 Materials and methods

This systematic review and meta-analysis was conducted in accordance with the Preferred Reporting Items for Systematic Reviews and Meta-Analyses (PRISMA) statement (16).

### 2.1 Search strategy

Two independent investigators conducted a systematic literature search of PubMed, Web of Science, Embase, Cochrane Library, China National Knowledge Infrastructure (CNKI), Wanfang Data, and Viper Information Products (VIP) databases. The search was conducted using a combination of subject terms and free words. The English search terms were as follows: “goal-directed/goal oriented/target-directed/mobilization/mobilisation/activity/rehabilitation/exercise/Early Ambulation” “Intensive Care Units/Critical Care/Respiration Artificial/intensive care unit/ICU/critical care/mechanical ventilation” “randomized controlled trial/randomized/randomised/RCT.” Additionally, the reference lists of the retrieved articles were manually searched to identify additional relevant studies. The search covered the period from the inception of each database to November 30, 2025. The complete search strategies for all databases, including search terms, field restrictions, search dates, and the number of records retrieved, are provided in Supplementary Table S2.

### 2.2 Inclusion and exclusion criteria

Inclusion criteria: adult ICU patients (≥18 years); EGDM program (daily individualized goal-setting, structured assessment [e.g., ICU Mobility Scale], multidisciplinary team [physicians, nurses, rehabilitation therapists]); control group receiving usual care (vital sign monitoring, bed positioning, passive range of motion by nurses, out-of-bed activities per physician orders); primary outcomes: incidence of ICU-AW and MRC sum score; secondary outcomes: mechanical ventilation duration, ICU length of stay, and Barthel Index; randomized controlled trials. Studies were considered eligible as RCTs only when randomized group allocation could be verified from the full-text Methods. Studies using non-random or quasi-random allocation procedures, such as allocation according to admission time, were considered not to meet the RCT design criterion.

Exclusion criteria: duplicate publications; studies with inaccessible or unextractable data; studies not published in Chinese or English.

### 2.3 Data extraction

Two researchers who had received training in evidence-based nursing methodology independently performed the literature search and screening. Retrieved records were imported into Zotero reference management software, and duplicate records were removed using its automatic deduplication function. Two researchers independently screened the titles and abstracts to exclude obviously irrelevant studies. The full texts of the remaining articles were then reviewed to determine final eligibility. When data were missing or reporting was unclear, the corresponding authors were contacted in an attempt to obtain the necessary information. Two researchers independently extracted the data and cross-checked the results. Any disagreements were resolved through discussion or arbitration by a third researcher or through discussion. A standardized data extraction form was used to collect study characteristics, including first author, year of publication, country, sample size, patient characteristics, intervention and control characteristics, and outcome data. For EGDM interventions, additional information on intervention implementation was extracted based on the key domains recommended by the Template for Intervention Description and Replication (TIDieR) checklist, including intervention components, mode of delivery, intervention providers, timing of initiation, progression strategies, safety criteria, and intervention duration. When specific intervention details were insufficiently reported in the original studies, the information was recorded as not reported. For studies reporting ICU-acquired weakness, we additionally extracted the study-level definition, assessment instrument, diagnostic threshold, assessment timing, and assessor/blinding information where available. These details are summarized in Supplementary Table S3.

### 2.4 Quality assessment of literature

Two researchers trained in evidence-based methodology assessed the risk of bias in the included RCTs using the Risk of Bias 2.0 (RoB 2.0) tool recommended by the Cochrane Collaboration (17, 18). RoB 2 assessment was performed only after study eligibility had been established. This tool evaluates bias across five domains: bias arising from the randomization process, bias due to deviations from intended interventions, bias due to missing outcome data, bias in measurement of the outcome, and bias in selection of the reported result. Each domain was judged as “low risk of bias,” “some concerns,” or “high risk of bias.” Any disagreements were resolved through discussion or arbitration by a third researcher or through discussion. The results of the risk-of-bias assessment were used to interpret the reliability of the meta-analysis findings and were considered in the subgroup analyses and sensitivity analyses.

### 2.5 Certainty of evidence

The certainty of evidence for each outcome was assessed using the Grading of Recommendations Assessment, Development and Evaluation (GRADE) approach. Randomized controlled trials were initially considered high-certainty evidence and were assessed across five domains: risk of bias, inconsistency, indirectness, imprecision, and publication bias. Based on these assessments, the certainty of evidence was categorized as high, moderate, low, or very low. The GRADE evidence profile was generated using GRADEpro GDT (19).

### 2.6 Statistical analysis

The meta-analysis was performed using RevMan 5.4 software. The risk ratio (*RR*) was used as the effect measure for dichotomous variables, and the mean difference (*MD*) was used for continuous variables, each presented with its 95% confidence interval (*CI*). Heterogeneity was assessed using the I^2^ statistic. If *I^2^* < 50% and *p* > 0.1, a fixed-effects model was applied; if *I^2^* ≥ 50% or *p* ≤ 0.1, a random-effects model was applied, and subgroup analyses or sensitivity analyses were conducted to explore potential sources of heterogeneity (20). For the primary outcomes, a subgroup analysis was prespecified by allocation concealment. When heterogeneity was high, sensitivity analyses were performed by excluding outlier studies or restricting the analysis to high-quality studies to test the robustness of the results.

## 3 Results

### 3.1 Literature search results

A total of 1,263 records were identified through the initial search. After duplicate removal, 1,065 records remained. Following title and abstract screening, 23 articles were assessed for full-text eligibility. Of these, 14 articles were excluded because they did not meet the RCT design criterion after full-text assessment of the reported allocation procedures (*n* = 7), data errors (*n* = 2), or unavailable full-text reports (*n* = 5). Finally, 9 RCTs (15, 21–28) were included, comprising 4 English and 5 Chinese studies and involving a total of 907 patients. The study selection process is presented in Figure 1. Details of the 14 full-text articles excluded after eligibility assessment and the reasons for exclusion are provided in Supplementary Table S1.

**Figure 1:**
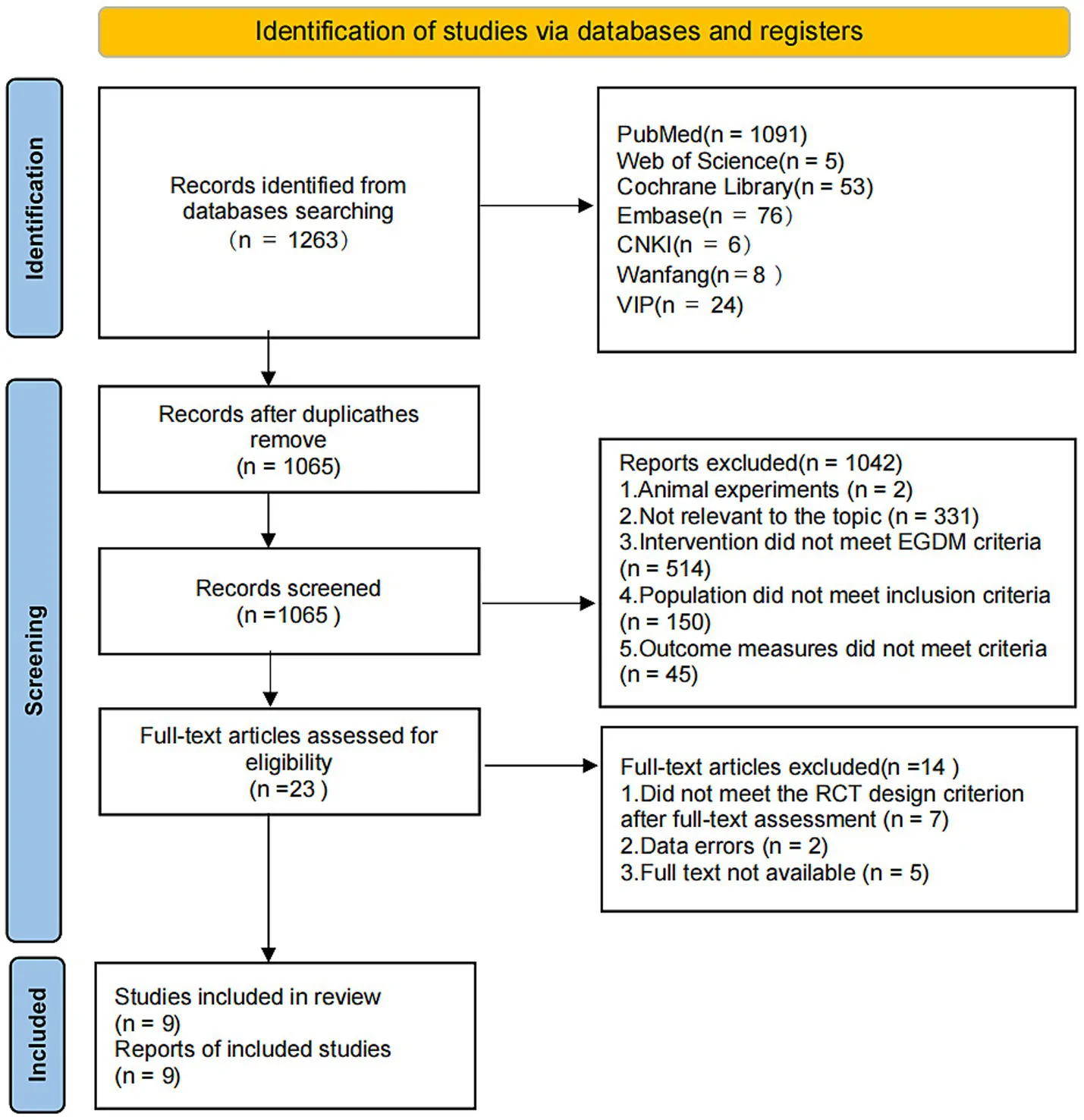
Flowchart of literature selection.

### 3.2 Basic characteristics and quality evaluation results

The basic characteristics of the nine included RCTs (15, 21–28) are presented in Table 1. Although all included studies met the predefined operational definition of EGDM, variation was observed in intervention implementation. Despite differences in timing, frequency, intensity, and progression, all interventions shared the three predefined core components of structured assessment, individualized goal setting, and multidisciplinary delivery. They were therefore considered conceptually comparable at the intervention-framework level rather than identical in implementation. Specifically, the included studies differed in ICU populations, including general ICU patients, surgical ICU patients, mechanically ventilated patients, and patients after acute type A aortic dissection surgery. The timing, progression strategies, and delivery of mobilization also varied across studies. Some studies applied IMS- or SOMS-based graded progression, whereas others used individualized rehabilitation protocols or multidisciplinary activity programs. In addition, the reported intervention duration and safety criteria were not consistently described among studies.

**Table 1:** Characteristics of the included studies.

Author	Year	Area	Patient characteristics	Age (MD)		Sample size I/C (*n*)	Intervention		Outcome
				Experimental group	Control group		EGDM intervention characteristics	Control group	
Hodgson (21)	2016	Australia, New Zealand	Mechanically ventilated >24 h	64.00	53.00	29/21	Assessment: IMS and physiological criteria; Goal setting: IMS-based maximal tolerable activity goals; Provider: activity team (physiotherapist, assistant, and nurse); Progression: increasing duration according to IMS level; Safety criteria: cardiovascular and respiratory stability criteria; Duration: 30/45/60 min/day according to IMS level; Initiation: day of enrolment (median 3 days after ICU admission in both groups); Prescribed dose: IMS 1–2, 30 min/day; IMS 4–6, 45 min/day; IMS 7–10, 60 min/day; Delivered/achieved dose: median 20 min/day (IQR 0–40) vs. 7 min/day (IQR 0–15) in control.	Routine rehabilitation	①②③④
Lin (22)	2023	China	Patients after acute type A aortic dissection surgery	53.40	52.30	39/38	Assessment: IMS, RASS, and respiratory/circulatory assessment; Goal setting: IMS-based progressive goals; Provider: multidisciplinary team; Progression: stepwise progression (maximum one level/day); Safety criteria: interruption when SBP ≥ 180 mmHg or DBP ≥ 90 mmHg; Duration: NR. Initiation: 48 h after surgery; Prescribed dose: no fixed daily minute-based dose reported; activity prescribed according to IMS level, with ≥4 h between two exercise sessions; Delivered/achieved dose: NR.	Routine rehabilitation	①②③④⑤
Schaller (15)	2016	Austria, Germany, United States	Surgical ICU patients, mechanically ventilated <48 h	66.00	64.00	104/96	Assessment: SOMS-based assessment; Goal setting: mobility-level goals; Provider: interprofessional team; Progression: algorithm-based progression; Safety criteria: predefined safety criteria; Duration: NR. Initiation: no later than 1 day after trial enrolment; Prescribed dose: no fixed daily duration; daily SOMS target level 0–4; Delivered/achieved dose: daily target achieved on 817/918 intervention days (89%).	Routine rehabilitation	①④⑤
McWilliams (23)	2018	United Kingdom	Mechanically ventilated ≥5 days	62.00	61.00	52/50	Assessment: MMS with SOFA/APACHE/Charlson assessment; Goal setting: individualized goals updated weekly based on MMS; Provider: specialized rehabilitation team with MDT input; Progression: function-oriented progression; Safety criteria: predefined safety criteria with no intervention-related SAE; Duration: mean 35–38 min/day. Initiation: NR; Prescribed dose: no fixed daily minute-based dose; structured goal management with weekly goal setting and updating; Delivered/achieved dose: mean treatment duration 38.3 min/day vs. 35.4 min/day in control; active rehabilitation 64% vs. 51%.	Routine rehabilitation	②③④⑤
Bian Hong (24)	2022	China	Mechanically ventilated ≥24 h	67.93	64.83	28/28	Assessment: IMS/RASS/CPOT; Goal setting: graded goals (0–4 levels) with reminder cards; Provider: multidisciplinary team and trained nurses; Progression: stepwise progression; Safety criteria: HR/BP/SpO₂ monitoring with interruption for intolerance; Duration: 10–20 min/session, 1–3 times/day. Initiation: NR; Prescribed dose: passive joint exercise 10 min/session, 3 times/day; cycle ergometry 15–20 min/session, twice daily; sitting training 15–20 min/session, 1–3 times/day; Delivered/achieved dose: NR.	Routine rehabilitation	②③④
He Yan (25)	2024	China	Mechanically ventilated ≥24 h	64.59	63.74	47/47	Assessment: Comprehensive assessment including oxygenation and vital signs; Goal setting: graded goals (0–4 levels); Provider: EGDM team and trained nurses; Progression: stepwise progression with reassessment before ventilator weaning; Safety criteria: vital sign monitoring under light sedation; Duration: 10–20 min/session, 2–3 times/day. Initiation: after 24 h of mechanical ventilation, when predefined clinical stability criteria were met; Prescribed dose: passive joint exercise 10 min/session, 3 times/day; cycle ergometry 15 min/session, twice daily; bedside sitting 15 min/session, twice daily; standing/walking 15–20 min/session, twice daily; Delivered/achieved dose: NR.	Routine rehabilitation	①②③④⑤
Gu Jia (26)	2023	China	ICU stay ≥24 h	58.70	56.62	75/75	Assessment: IMS/RASS/CPOT; Goal setting: five-level individualized goals; Provider: multidisciplinary team; Progression: stepwise progression; Safety criteria: interruption for intolerance or respiratory/muscle instability; Duration: 20–60 min/session, twice daily. Initiation: exact time point NR; intervention was implemented after safety screening in patients with ICU stay ≥24 h; Prescribed dose: five-level protocol, approximately 20–60 min/session with level-specific frequency (generally twice daily); Delivered/achieved dose: NR.	Routine rehabilitation	①②③⑤
Jin Yaqi (27)	2023	China	Mechanically ventilated patients	59.60	62.20	40/40	Assessment: IMS and safety assessment; Goal setting: five-level rehabilitation goals; Provider: multidisciplinary team; Progression: stepwise progression; Safety criteria: predefined initiation and interruption criteria (HR/BP/RR/SpO₂/FiO₂/PEEP/vasoactive medication); Duration: 15 min to >60 min/session, 1–3 times/day. Initiation: NR; Prescribed dose: Level 1, 15 min/session twice daily; Level 2, 30 min active exercise + 30–60 min balance training; Level 3, 20–30 min resistance exercise + 30–60 min sitting-up training; Levels 4–5, >60 min/session (Level 5 twice daily); Delivered/achieved dose: four interruption events in the intervention group; total delivered duration NR.	Routine rehabilitation	②③④
Ma Rongrong (28)	2023	China	Mechanically ventilated ≥24 h	63.05	62.19	49/49	Assessment: IMS and safety parameters (HR/BP/RR/SpO₂/FiO₂/PEEP); Goal setting: five-level goals based on IMS; Provider: multidisciplinary management team; Progression: IMS-based graded progression; Safety criteria: predefined initiation, tolerance, and interruption criteria; Duration: 15–50 min/session plus balance training, 1–2 times/day. Initiation: NR; Prescribed dose: passive exercise + sitting 15–30 min; active-assisted/antigravity exercise 30 min/session; active exercise 30 min/session + balance training 45 min/session; walking 45 min/session + cycle ergometry 30 min/session; Delivered/achieved dose: NR.	Routine rehabilitation	②③④

All included studies were randomized controlled trials. The control group received conventional rehabilitation measures, while the intervention group received the EGDM protocol. Initiation refers to the reported time point at which the EGDM intervention was started. Prescribed dose refers to the planned session duration and/or frequency specified in the intervention protocol, whereas delivered/achieved dose refers to the actual mobilization exposure reported by the study. NR indicates not reported. Outcome measures included: ① Incidence of ICU-AW; ② Medical Research Council (MRC) sum score; ③ Duration of mechanical ventilation; ④ ICU length of stay; ⑤ Barthel Index.

The results of the risk of bias assessment (Figure 2) showed that for the randomization process four studies (15, 22, 23, 27) were rated as low risk of bias, while the remaining five studies (21, 24–26, 28) were judged as having some concerns. For deviations from intended interventions, one study (28) was judged as low risk of bias, while the remaining eight studies (15, 21–27) were judged as having some concerns. All nine studies (15, 21–28) were judged as having a low risk of bias for missing outcome data. For measurement of the outcome, six studies (15, 21–24, 27) were judged as having a low risk of bias, while the remaining three studies (25, 26, 28) were judged as having some concerns. All nine studies (15, 21–28) were judged as having a low risk of bias for selection of the reported result. Overall, two studies (15, 27) were judged as having a low risk of bias, while the remaining seven studies (21–26, 28) were judged as having some concerns. Except for Hodgson (21), all included studies compared baseline characteristics such as age, sex, body mass index, and disease severity between groups, and reported no major baseline imbalances between the intervention and control groups. Hodgson (21) reported differences in some baseline indicators, but the authors adjusted for these in subsequent analyses. The basic characteristics of the included studies are presented in Table 1, and the risk of bias assessment results are shown in Figure 2.

**Figure 2:**
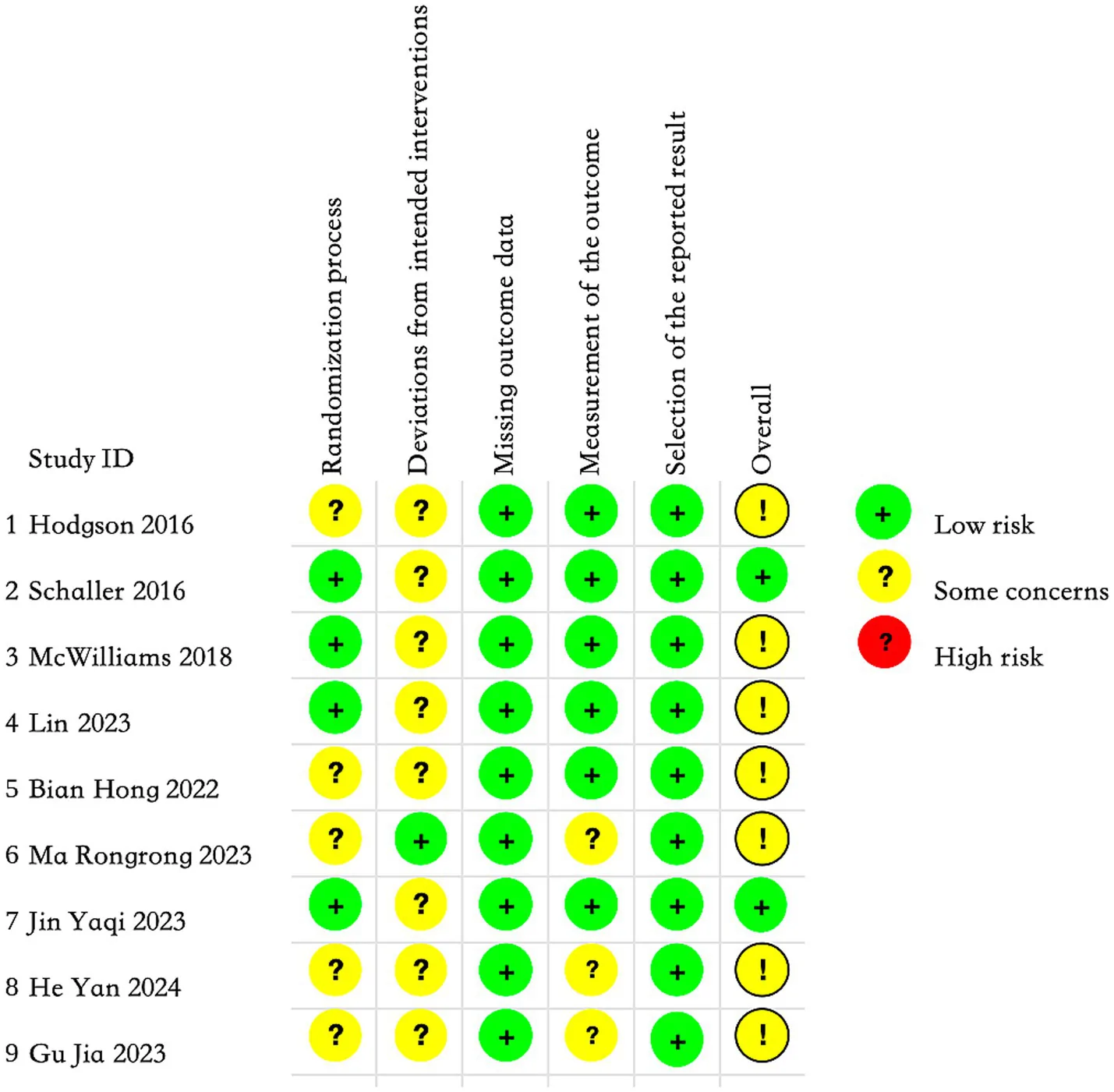
Risk of bias summary graph.

It is important to acknowledge that, given the nature of the intervention (early goal-directed mobilization), blinding of participants and personnel was not feasible in most studies, which may have contributed to some concerns regarding deviations from intended interventions. However, the risk of bias related to outcome measurement varied across studies. Therefore, these methodological limitations should be considered when interpreting the findings.

### 3.3 Meta-analysis results

Meta-analyses were performed for outcomes reported by at least 2 studies, including the incidence of ICU-AW, MRC sum score, mechanical ventilation duration, ICU length of stay, and Barthel Index.

#### 3.3.1 Incidence of ICU-AW

Five studies (15, 21, 22, 25, 26) reported the incidence of ICU-AW. Study-level definitions and ascertainment methods for ICU-AW are summarized in Supplementary Table S3. The pooled analysis using a random-effects model showed that EGDM may reduce the risk of ICU-AW [*RR* = 0.57, 95%*CI* (0.33 ~ 0.97)*, p* = 0.04 for the overall effect], although heterogeneity was high (*I^2^* = 77%, *p* = 0.002 for heterogeneity) (Figure 3). Subgroup analysis by allocation concealment revealed that, in the subgroup with adequate allocation concealment (15, 21, 22), the pooled effect was not statistically significant [*RR* = 0.71, 95%*CI* (0.40 ~ 1.24), *p* = 0.23 for the overall effect; *I^2^* = 67%, *p* = 0.05 for heterogeneity], and heterogeneity was lower than that in the main analysis. In contrast, in the subgroup without adequate allocation concealment (25, 26), the pooled effect was statistically significant and larger in magnitude [*RR* = 0.43, 95%*CI* (0.27 ~ 0.68), *p* = 0.0004 for the overall effect; *I^2^* = 0%, *p* = 0.67 for heterogeneity]. The test for subgroup differences was not statistically significant (*p* = 0.17 for subgroup differences) (Figure 4). Given the differences in methodological quality among the studies included in the main analysis, a sensitivity analysis was performed by sequentially excluding individual studies. The results showed that when only the two most methodologically rigorous international multicenter RCTs (15, 21) were included, the effect of EGDM on the incidence of ICU-AW was not statistically significant [*RR* = 0.84, 95%*CI* (0.48 ~ 1.46), *p* = 0.54 for the overall effect; *I^2^* = 55%, *p* = 0.13 for heterogeneity] (Figure 5).

**Figure 3:**
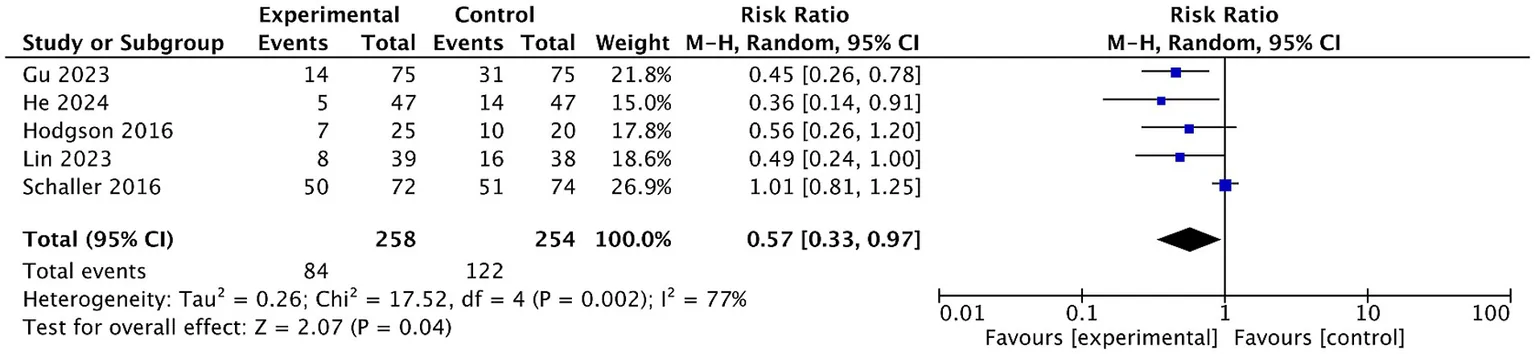
Meta-analysis of the effect of EGDM on the incidence of ICU-AW.

**Figure 4:**
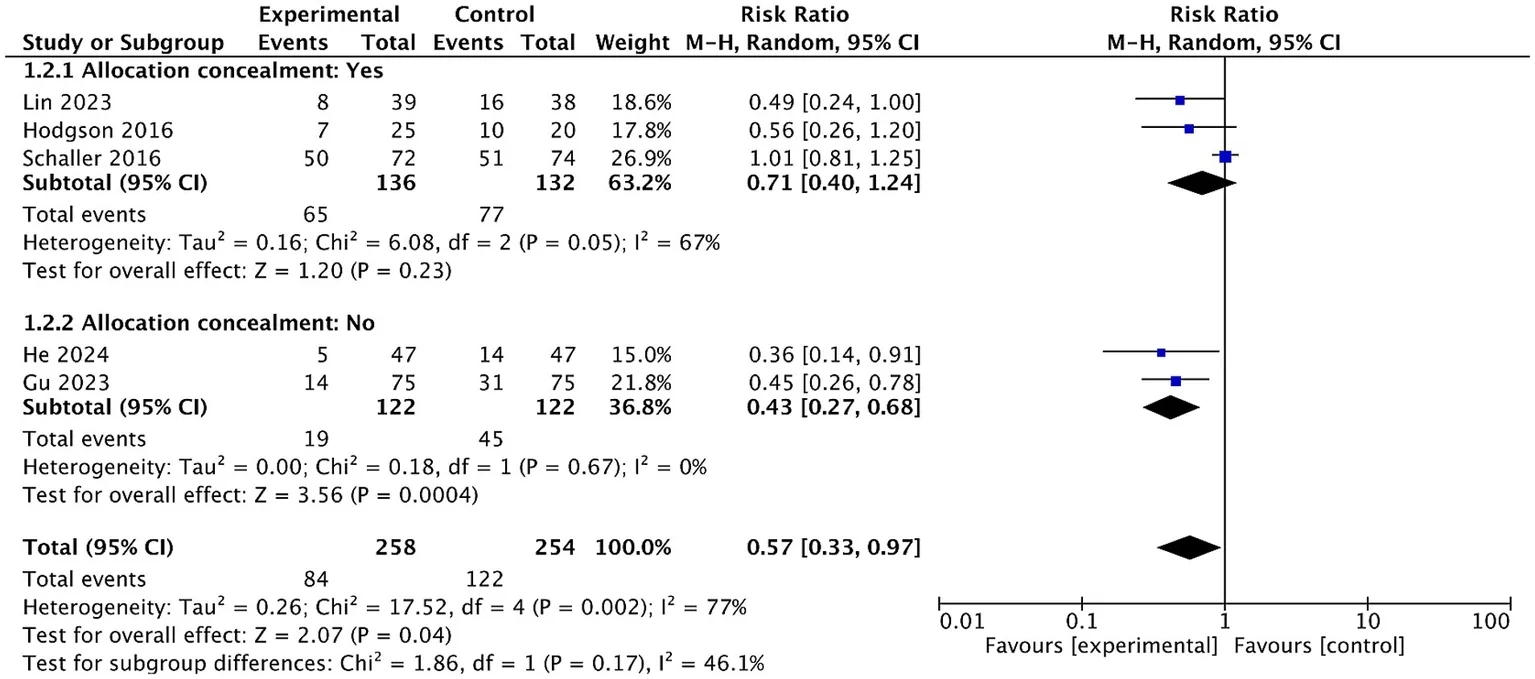
Subgroup analysis of the effect of EGDM on the incidence of ICU-AW.

**Figure 5:**
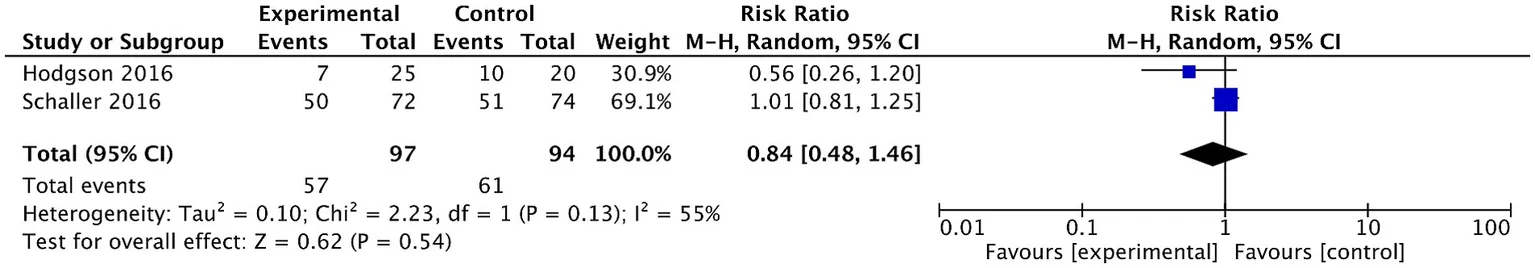
Sensitivity analysis of the effect of EGDM on the incidence of ICU-AW.

#### 3.3.2 MRC sum score

Seven studies (21–23, 25–28) reported muscle strength measured using the MRC sum score. The pooled analysis (Figure 6) showed that EGDM significantly improved muscle strength [*MD* = 4.94 points, 95%*CI* (3.39 ~ 6.50), *p* < 0.00001 for the overall effect; *I^2^* = 59%, *p* = 0.02 for heterogeneity]. Subgroup analysis by allocation concealment (Figure 7) showed that the pooled effect sizes were similar between the subgroup with adequate allocation concealment (21, 22, 27) [*MD* = 5.25 points, 95%*CI* (3.44 ~ 7.06), *p* < 0.00001 for the overall effect; *I^2^* = 0%, *p* = 0.61 for heterogeneity] and the subgroup without adequate allocation concealment (23, 25, 26, 28) [*MD* = 4.64 points, 95%*CI* (2.25 ~ 7.03), *p* = 0.0001 for the overall effect; *I^2^* = 77%, *p* = 0.004 for heterogeneity], and the test for subgroup differences was not statistically significant (*p* = 0.69 for subgroup differences). Although results varied considerably among studies with lower methodological quality, there was no consistent evidence that lower methodological quality systematically overestimated or underestimated the effect of EGDM on muscle strength.

**Figure 6:**
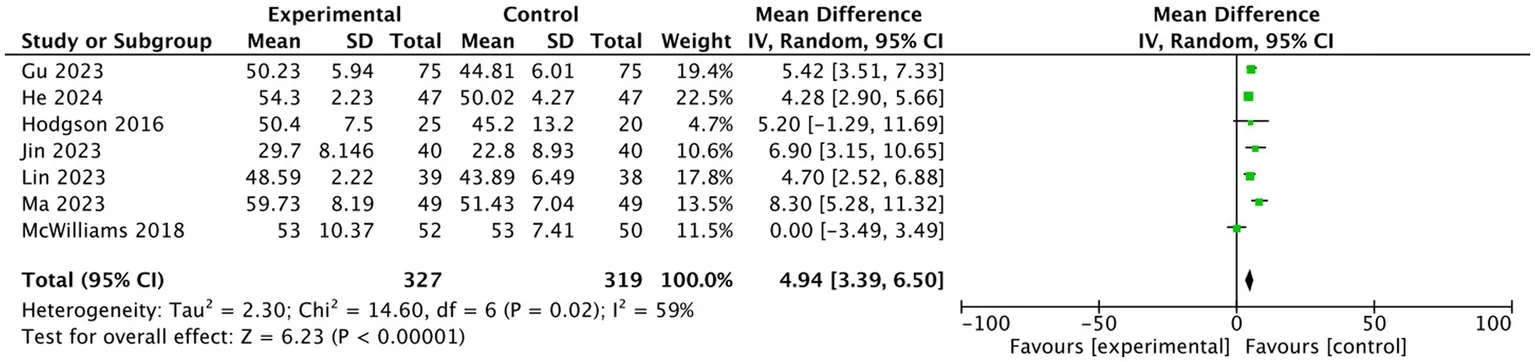
Meta-analysis of the effect of EGDM on MRC sum score.

**Figure 7:**
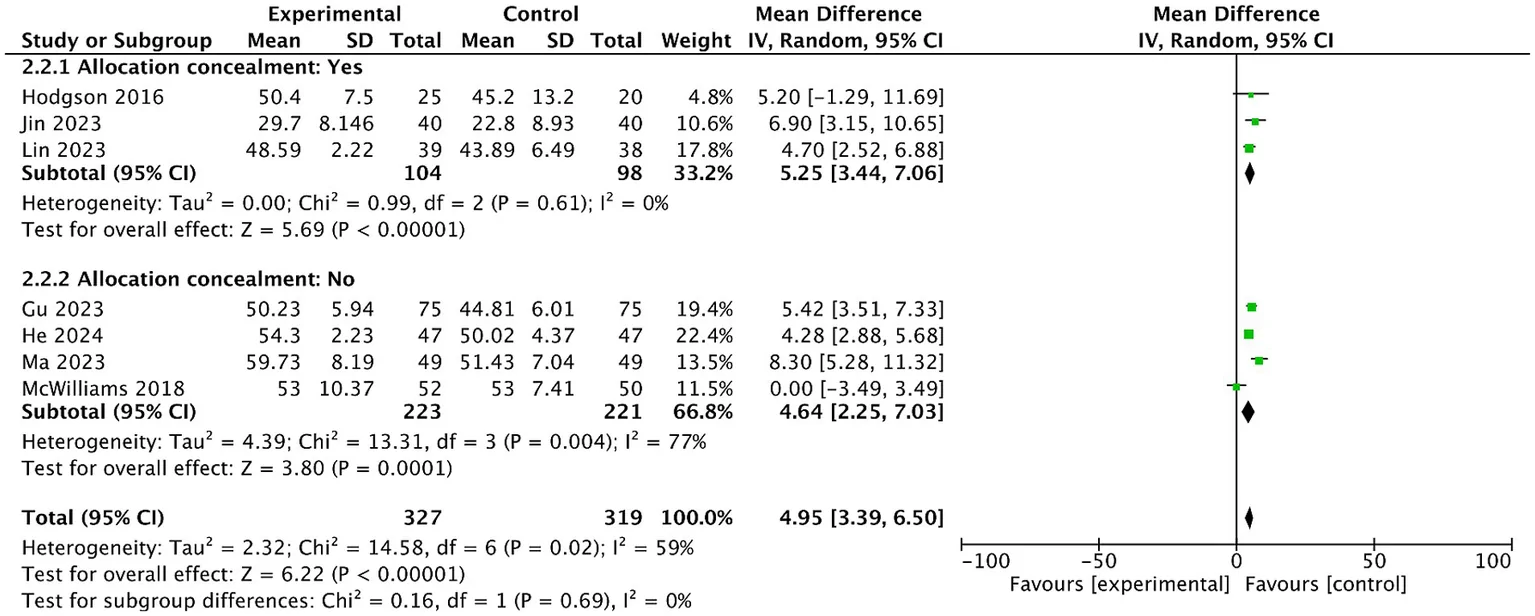
Subgroup analysis of the effect of EGDM on MRC sum score.

#### 3.3.3 Mechanical ventilation duration

Nine studies (15, 21–28) reported the duration of mechanical ventilation. The pooled analysis (Figure 8) showed that EGDM might shorten the duration of mechanical ventilation [*MD* = -81.95 h, 95%*CI* (−115.45 ~ −48.46), *p* < 0.00001 for the overall effect], although heterogeneity was extremely high (*I^2^* = 85%, *p* < 0.00001 for heterogeneity). To explore the sources of heterogeneity, a sensitivity analysis was first performed by sequentially excluding individual studies. The results showed that even after excluding the two studies with the most extreme effect sizes (27, 28), substantial heterogeneity remained among the remaining seven studies (*I^2^* = 71%, *p* = 0.004 for heterogeneity), suggesting that heterogeneity was not attributable to a single study. Subsequently, a prespecified subgroup analysis by allocation concealment was conducted (Figure 9). The results showed that the difference in effect sizes between the subgroup with adequate allocation concealment (15, 21, 22, 27) and the subgroup without adequate allocation concealment (23–26, 28) was not statistically significant (*p* = 0.45 for subgroup differences), and both subgroups demonstrated high within-group heterogeneity (*I^2^* = 78%, *p* = 0.003 for heterogeneity; *I^2^* = 84%, *p* < 0.0001 for heterogeneity, respectively). These findings suggest that differences in allocation concealment were unlikely to be the primary source of heterogeneity.

**Figure 8:**
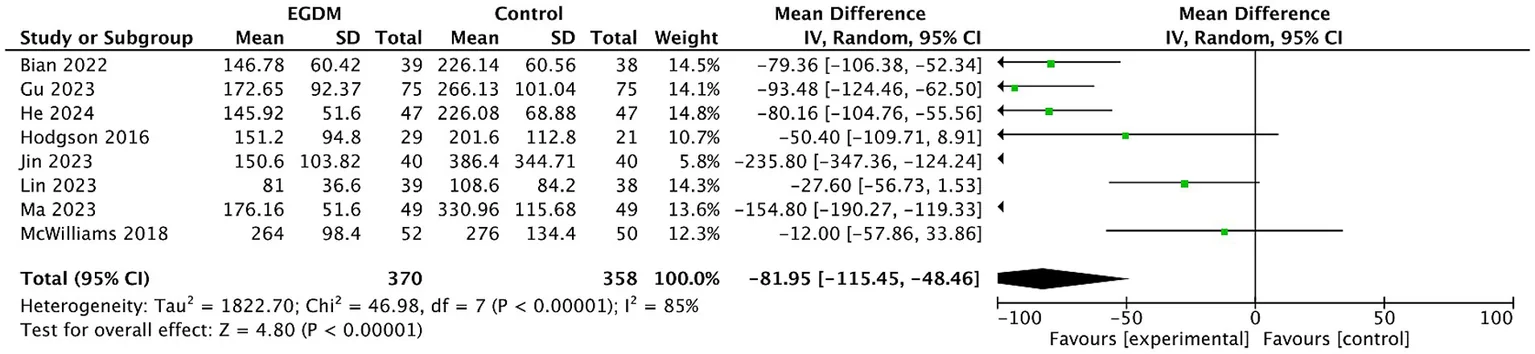
Meta-analysis of the effect of EGDM on duration of mechanical ventilation.

**Figure 9:**
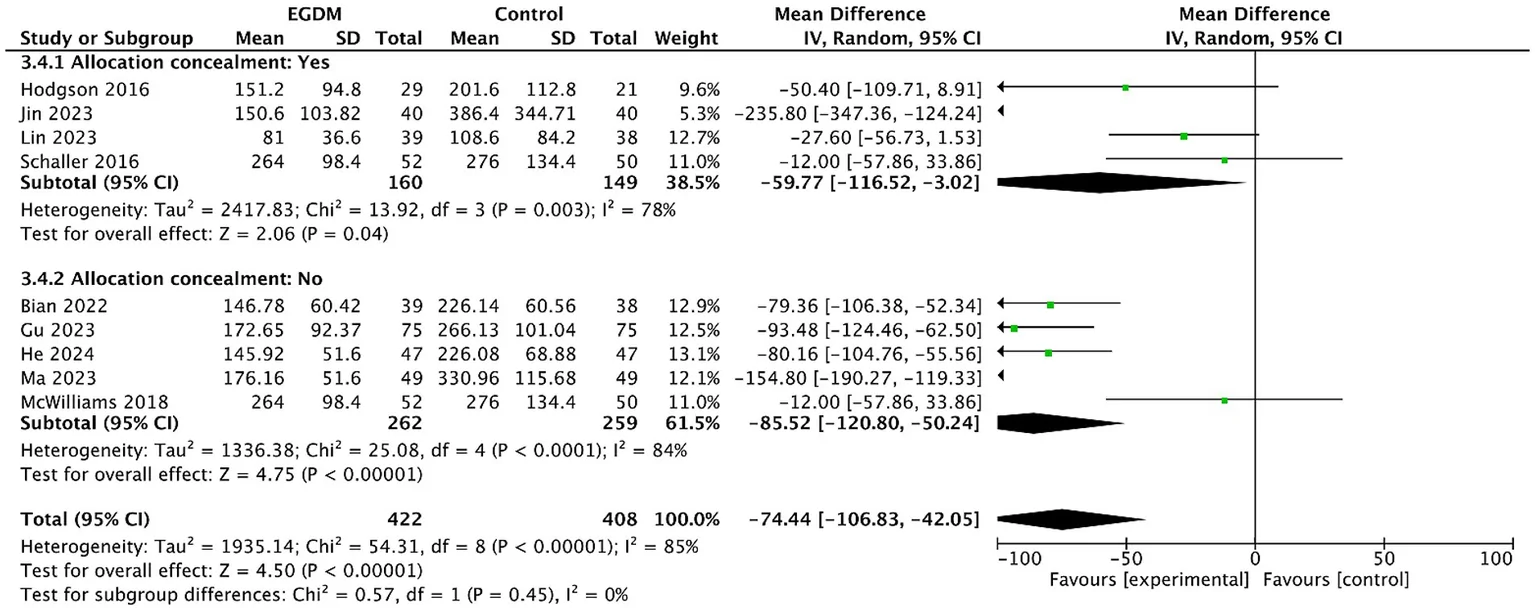
Subgroup analysis of the effect of EGDM on duration of mechanical ventilation.

#### 3.3.4 ICU length of stay

Eight studies (15, 21–25, 27, 28) reported ICU length of stay. The pooled analysis (Figure 10) showed that EGDM might shorten ICU length of stay [*MD* = -4.55 days, 95%*CI* (−6.22 ~ −2.89), *p* < 0.00001 for the overall effect], although heterogeneity was high (*I^2^* = 74%, *p* = 0.0004 for heterogeneity). A sensitivity analysis (Figure 11) was performed by sequentially excluding individual studies. After excluding the study by Jin (27), in which the control group had an exceptionally long ICU stay, heterogeneity decreased to 53% (*p* = 0.05 for heterogeneity), and the effect size became more conservative [*MD* = -3.96 days, 95%*CI* (−5.19 ~ −2.72), *p* < 0.00001 for the overall effect], while remaining statistically significant, supporting the robustness of the finding.

**Figure 10:**
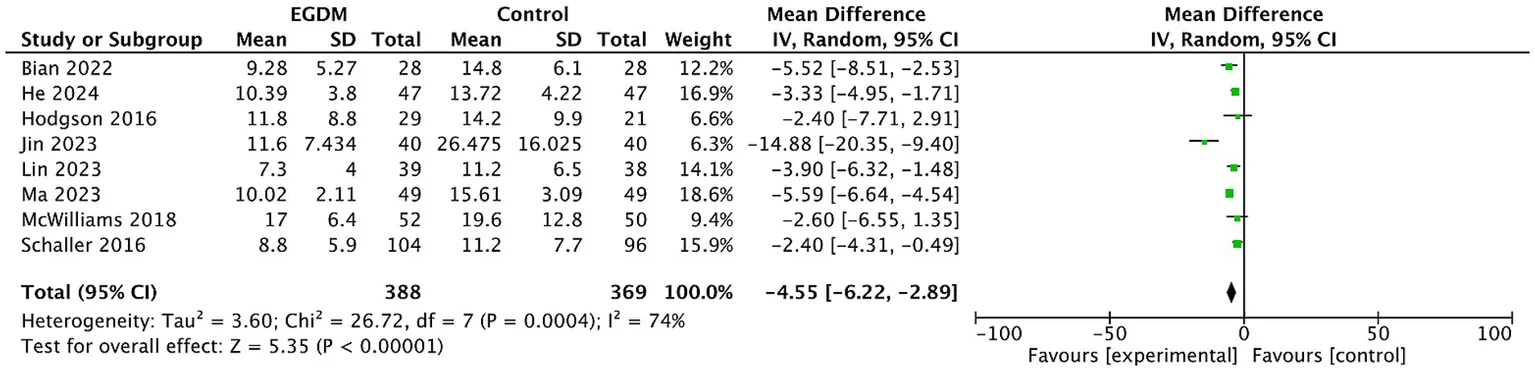
Meta-analysis of the effect of EGDM on length of ICU stay.

**Figure 11:**
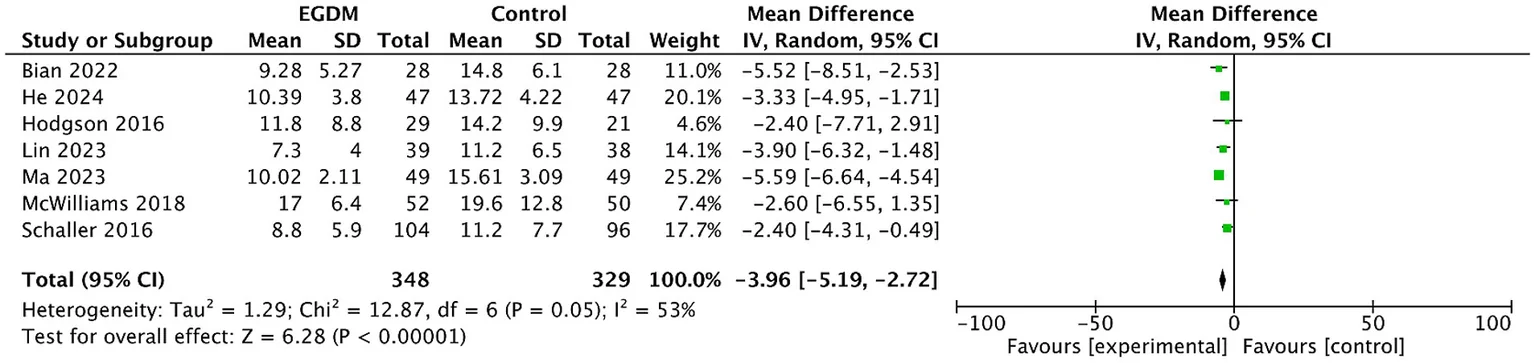
Sensitivity analysis of the effect of EGDM on length of ICU stay.

#### 3.3.5 Barthel index

Four studies (22, 23, 25, 26) reported the Barthel Index at ICU discharge or hospital discharge. The primary analysis including all four studies (Figure 12) showed no statistically significant difference between groups [*MD* = 5.16 points, 95%*CI* (−1.03 ~ 11.35), *p* = 0.10 for the overall effect], although heterogeneity was extremely high (*I^2^* = 89%, *p* < 0.00001 for heterogeneity). Further examination suggested that the heterogeneity was mainly attributable to the study by McWilliams (23), in which the control group had a baseline Barthel Index score of 90 out of 100, suggesting a potential ceiling effect. In a sensitivity analysis excluding this study (Figure 13), heterogeneity among the remaining three studies (22, 25, 26) decreased to *I^2^* = 18% (*p* = 0.30 for heterogeneity), and the pooled results showed that EGDM significantly improved functional status in patients with moderate baseline function [*MD* = 8.27 points, 95%*CI* (5.66 ~ 10.87), *p* < 0.00001 for the overall effect].

**Figure 12:**
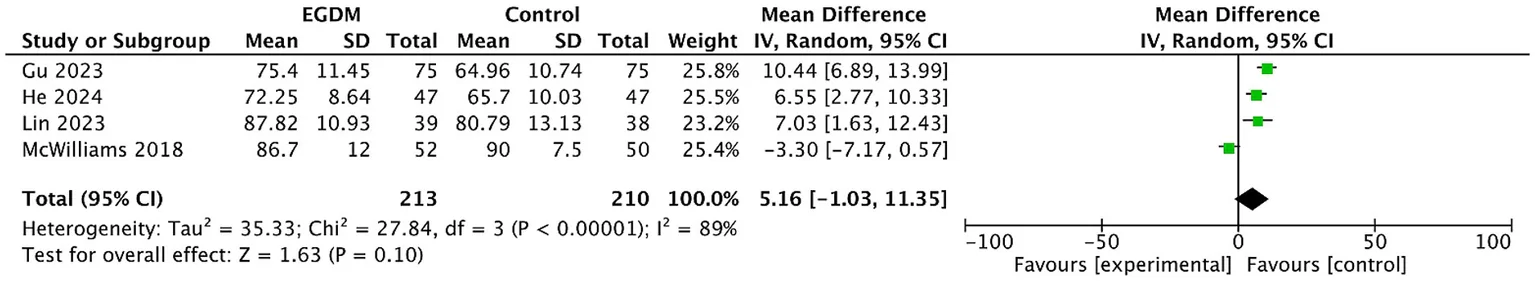
Meta-analysis of the effect of EGDM on Barthel Index.

**Figure 13:**
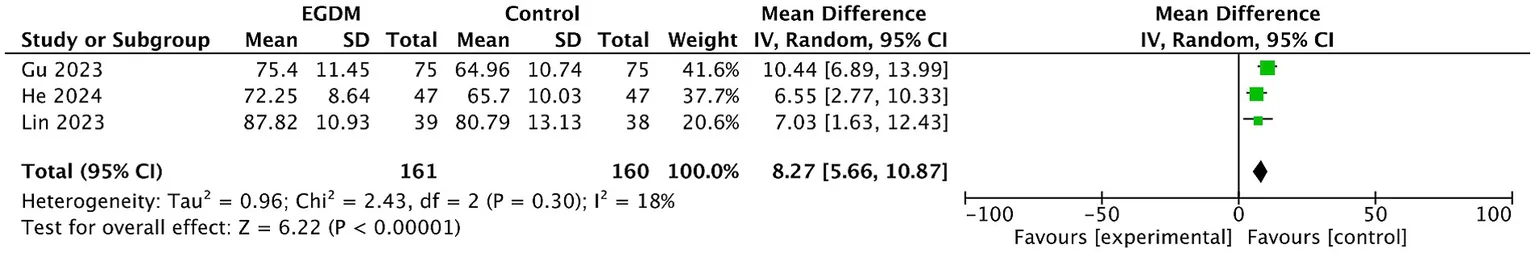
Sensitivity analysis of the effect of EGDM on Barthel Index.

### 3.4 Certainty of evidence

The GRADE assessment of the five outcomes is presented in Table 2. The certainty of evidence was rated as moderate for the MRC sum score and ICU length of stay, and low for the incidence of ICU-AW, the duration of mechanical ventilation, and the Barthel Index. The main reasons for downgrading the certainty were concerns regarding risk of bias, inconsistency, and imprecision.

**Table 2:** GRADE assessment of the certainty of evidence for each outcome.

Certainty assessment							№ of patients		Effect		Certainty	Importance
№ of studies	Study design	Risk of bias	Inconsistency	Indirectness	Imprecision	Other considerations	The incidence of ICU-AW	Placebo	Relative (95% CI)	Absolute (95% CI)		
ICU-AW incidence												
5	Randomised trials	Serious	Serious	Not serious	Not serious	None	84/258 (32.6%)	122/254 (48.0%)	**RR 0.57** (0.33 to 0.97)	**207 fewer per 1,000** (from 322 fewer to 14 fewer)	⨁⨁◯◯ Low	
Muscle strength (MRC sum score)												
7	Randomised trials	Serious	Not serious	Not serious	Not serious	None	327	319	-	MD 4.94 higher (3.39 higher to 6.5 higher)	⨁⨁⨁◯ Moderate	
Mechanical ventilation duration												
9	Randomised trials	Serious	Serious	Not serious	Not serious	None	370	358	-	MD 81.95 lower (115.45 lower to 48.46 lower)	⨁⨁◯◯ Low	
ICU length of stay												
8	Randomised trials	Serious	Not serious	Not serious	Not serious	None	388	369	-	MD 4.55 lower (6.22 lower to 2.89 lower)	⨁⨁⨁◯ Moderate	
Barthel index												
4	Randomised trials	Serious	Not serious	Not serious	Serious	None	213	210	-	MD 5.16 higher (1.03 lower to 11.35 higher)	⨁⨁◯◯ Low	

CI, confidence interval; MD, mean difference; RR, risk ratio.

## 4 Discussion

This meta-analysis systematically evaluated the multifaceted effects of EGDM on ICU patients, with particular attention to methodological quality. The results demonstrated that EGDM effectively improved muscle strength and certain functional outcomes; however, a reduction in the incidence of ICU-acquired weakness was not confirmed by the available high-quality studies. Furthermore, the substantial heterogeneity observed across multiple outcomes suggests that the intervention effects may be influenced by clinical and contextual factors.

### 4.1 Effect of EGDM on the incidence of ICU-AW

This study found that the estimated effect of EGDM on the incidence of ICU-AW varied according to the methodological quality of the included studies. Although the initial pooled analysis showed a trend toward risk reduction (*RR* = 0.57), heterogeneity among the included studies was high (*I^2^* = 77%). In the sensitivity analysis restricted to the two most methodologically rigorous high-quality RCTs (15, 21), the effect was not statistically significant.

Potential sources of heterogeneity include several factors. First, detection bias may be difficult to avoid. The diagnosis of ICU-AW relies on bedside clinical assessments such as the MRC sum score. Although Schaller (15) and Hodgson (21) made efforts to implement blinding, patients and intervention providers could not be blinded due to the nature of the intervention, which may have introduced bias. Moreover, some studies reporting positive findings did not report the implementation of blinding. Second, intervention intensity and timing may contribute to heterogeneity. ICU-AW is a syndrome involving the interplay of inflammation, metabolic disturbances, and neuromuscular dysfunction, reflecting a complex pathophysiological process (29). As a comprehensive rehabilitation intervention, EGDM varies considerably in intensity and frequency across studies and the intervention dose in some studies may have been insufficient to meaningfully influence the complex pathophysiology of ICU-AW. A meta-analysis by Luo et al. (30) found that initiating activity programs within 24 to 72 h after ICU admission may be associated with the greatest reduction in the incidence of ICU-AW. The protocols included in the present study may have differed in initiation timing, thereby affecting the overall effect. Third, heterogeneity in patient populations may also have contributed to the observed variability. The included studies differed in disease severity, underlying etiology, and ICU-AW risk factors among patients, which may have masked the effect of the intervention in specific subgroups (31).

Of note, neither of the two most methodologically rigorous high-quality RCTs (15, 21) demonstrated a significant effect of EGDM on the incidence of ICU-AW. This may be attributable to the following factors. First, statistical power may have been insufficient (32). Although the study by Schaller et al. (15) was a multicenter RCT, the incidence of ICU-AW was only a secondary outcome, and the sample size was not calculated specifically for this outcome. The study by Hodgson et al. (21) was a feasibility RCT involving only 50 patients, and its sample size was likely insufficient to detect a difference in ICU-AW incidence. Second, the control groups received relatively intensive usual rehabilitation. In Schaller et al. (15), the control group also received standardized management including goal-directed sedation, daily awakening, and spontaneous breathing trials, and each center provided physiotherapy and activity support to control patients according to routine practice. In Hodgson et al. (21), the control group received standard ICU rehabilitation, and routine ICU rehabilitation in Australia and New Zealand is already relatively well established, with serving as regular members of the multidisciplinary team (33). In Schaller et al. (15), the difference in SOMS (Surgical Optimal Mobility Score) activity scores between the intervention and control groups was only 0.7 points. In Hodgson et al. (21), the difference in daily active mobilization time between the two groups was only 13 min. Such a limited difference in mobilization dose may be insufficient to produce a detectable difference in the incidence of ICU-AW (34).

Therefore, clinicians may consider assessing baseline functional status with the Barthel Index early after ICU admission. The current evidence does not conclusively establish that EGDM prevents ICU-AW. Although sedation management, nutritional support, and early initiation of mobilization are clinically relevant considerations in critically ill patients, these factors were not directly evaluated as components or effect modifiers in the present meta-analysis. In particular, the optimal timing of EGDM initiation cannot be determined from the current findings. Previous evidence suggesting initiation within 72 h should therefore be regarded as contextual evidence rather than a conclusion derived from this meta-analysis (30).

### 4.2 Effect of EGDM on MRC sum score

Unlike the findings for ICU-AW, the results of this meta-analysis showed a consistent improvement in the MRC sum score with EGDM. Except for McWilliams (23), in which the control group had unusually high baseline muscle strength and no between-group difference was observed, all six remaining studies reporting this outcome (21, 22, 25–28) reported a favorable effect of EGDM. The pooled analysis showed that EGDM significantly improved muscle strength (*MD* = 4.94 points, *p* < 0.00001), and subgroup analysis indicated that the effect estimate did not significantly differ according to methodological quality (*p* = 0.69 for subgroup differences). However, improvement in the continuous MRC sum score should not be interpreted as equivalent to a reduction in the incidence of ICU-AW, as these are related but distinct outcomes.

These findings are consistent with previously published systematic reviews. Tipping et al. (35), in their systematic review of early rehabilitation activities in ICU patients, also found that early rehabilitation significantly improved muscle strength scores, with relatively consistent effects across studies. However, the magnitude of improvement in muscle strength varies across studies. Luo et al. (30) found that muscle strength recovery was associated with the timing of mobilization initiation, with the greatest benefit observed when activities were initiated within 24 to 72 h after ICU admission. Initiation that was either too early or too late may have compromised the intervention effect. This finding suggests that differences in the timing of initiation across the RCTs included in the present study may be an important factor influencing the magnitude of muscle strength improvement.

In the present study, the pooled improvement in the MRC sum score was 4.94 points. Previous research has defined the minimal clinically important difference (MCID) for the total MRC score as 4 points (36). The pooled estimate in our study (4.94 points) exceeded this reference threshold, suggesting that the improvement may be clinically meaningful. Although this improvement alone would not necessarily shift a patient from below the ICU-AW diagnostic threshold (<48 points) into the normal range, improvements in muscle strength remain an important foundation for functional recovery. Previous studies have also reported positive associations between improvements in muscle strength and functional status and quality of life after hospital discharge (37, 38). These findings support the potential clinical value of EGDM for improving muscle strength in ICU patients (39).

### 4.3 Effect of EGDM on mechanical ventilation duration

The present analysis showed that EGDM may shorten the duration of mechanical ventilation, although between-study heterogeneity was extremely high. All nine studies reporting this outcome (15, 21–28) showed effect estimates favoring shorter ventilation duration, with Jin (27) and Ma (28) reporting the largest effect sizes.

The high heterogeneity suggests that the duration of mechanical ventilation may be influenced by multiple clinical and practice-related factors. First, practice settings differed across studies. Sedation depth and analgesic strategies vary across ICUs and institutions. Under deep sedation, patients are often unable to participate in active mobilization, thereby limiting the intervention effect of EGDM (40). In contrast, with lighter sedation, patients can engage in active activities earlier, potentially facilitating ventilator weaning (41, 42). Second, baseline patient characteristics differed across studies. The disease severity, baseline pulmonary function, and the indication for mechanical ventilation may directly affect the difficulty of weaning and the potential benefit of the intervention. The exceptionally long ventilation duration in the control group of Jin (27) (386.4 h) illustrates the potential influence of such between-study differences.

### 4.4 Effect of EGDM on ICU length of stay

The present study found that EGDM may shorten ICU length of stay (*MD* = −4.55 days), although between-study heterogeneity was high (*I^2^* = 74%). After excluding the outlying study in the sensitivity analysis, the effect persisted but became more conservative. The observed trend toward shorter ICU length of stay was consistent across the eight studies reporting this outcome (15, 21–25, 27, 28). However, ICU length of stay is determined by multiple clinical and organizational factors. EGDM may indirectly affect ICU length of stay by shortening the duration of mechanical ventilation, reducing complications such as delirium, and improving patients’ functional status, thereby enabling patients to meet ICU discharge criteria earlier (22, 26). At the same time, ICU length of stay is also significantly affected by nonclinical factors, such as hospital bed turnover policies and family support (42–44), which may contribute to between-study variability. Therefore, the effect of EGDM on ICU length of stay should be interpreted cautiously and considered within the broader context of overall ICU care and discharge processes. Accordingly, for patients with moderate pre-ICU functional status who are expected to return to community living, EGDM may be considered to support recovery in activities of daily living and to inform rehabilitation goal setting and discharge planning.

### 4.5 Effect of EGDM on Barthel index (activities of daily living)

In the primary analysis including all four studies, EGDM was not associated with a statistically significant improvement in the Barthel Index. However, substantial heterogeneity was observed. Among the four studies reporting the Barthel Index (22, 23, 25, 26), the study by McWilliams (23) was excluded from the sensitivity analysis because the control group had an extremely high baseline functional status (ceiling effect), suggesting a ceiling effect. The remaining three studies (22, 25, 26) showed highly consistent results (*I^2^* = 18%), with all three favoring improvement in the Barthel Index among patients with moderate baseline functional status, with improvements ranging from 6.55 to 10.44 points. EGDM may improve muscle strength and endurance (45), and the enhanced physical function enables patients to better perform basic activities such as bed mobility, transfers, and walking—core components assessed by the Barthel Index. This functional improvement directly enhances patient independence and readiness for discharge, providing potential clinical value for patients with moderate baseline function.

Therefore, it is recommended that clinicians assess patients’ baseline functional status using the Barthel Index early after ICU admission (46). For patients with moderate baseline function (i.e., moderately dependent according to the Barthel Index), EGDM should be actively implemented, and improving the Barthel Index should be considered one of the rehabilitation goals. By enhancing muscle strength and endurance, EGDM promotes patients’ ability to independently perform basic activities such as bed mobility, transfers, and walking, thereby improving their discharge readiness and quality of life.

### 4.6 Limitations and implications

This study has several limitations. First, regarding publication bias, the number of high-quality RCTs meeting the strict definition of EGDM was limited, resulting in small sample sizes for some sensitivity analyses. A total of nine studies were included, which is insufficient to effectively test publication bias using funnel plots. Second, substantial clinical heterogeneity existed among the EGDM interventions. Although all included studies met the predefined operational definition of EGDM, the specific implementation strategies varied considerably, including the timing of mobilization initiation, progression methods, intervention duration, safety criteria, and delivery models. These variations may have influenced the magnitude of intervention effects across outcomes. However, because the included studies did not consistently report detailed intervention characteristics, such as mobilization dose and actual activity delivery, further subgroup analyses based on intervention intensity or implementation features were not feasible. Third, some studies had insufficient reporting of key methodological aspects such as allocation concealment and blinding. Fourth, a number of outcome measures exhibited high heterogeneity. Although subgroup analyses and sensitivity analyses were performed, the sources of heterogeneity could not be fully explained, limiting the precision of the effect estimates. Fifth, potential language, regional, and publication-related effects should be considered when interpreting the findings. Five of the nine included studies were published in Chinese, several of which had relatively small sample sizes and incomplete reporting of methodological details. The five Chinese studies were conducted in different regions and were published in different years, and no apparent overlap among study populations was identified. One of these studies was a master’s dissertation, whereas the others were journal articles. Differences in publication type, usual-care practices, rehabilitation resources, and clinical settings across studies may have contributed to between-study variability and may limit the generalizability of the pooled estimates. In addition, restricting the search to Chinese- and English-language publications may have introduced language bias and excluded relevant evidence published in other languages. Potential publication bias and small-study effects also cannot be excluded. Sixth, the included studies predominantly involved general or surgical ICU populations and mechanically ventilated patients rather than specifically neurological or neurocritical care populations. Although the outcomes examined, particularly muscle strength and functional recovery, are relevant to neurorehabilitation, neurological subgroups were not separately analyzed in the included studies. Therefore, the present findings should not be directly extrapolated to critically ill neurological patients, and further studies are needed to evaluate EGDM specifically within neurocritical care populations.

Although the included studies were not specifically conducted in neurocritical care populations, EGDM may have indirect relevance to neurorehabilitation because the outcomes examined included muscle strength, mobility, and functional recovery. However, the present findings should not be directly extrapolated to neurocritical care populations, and its implementation in these populations requires additional neurological and physiological assessment. In particular, mobilization decisions should account for level of consciousness, intracranial pressure and cerebral perfusion where relevant, hemodynamic and respiratory stability, and the presence of invasive neurological devices. Furthermore, the optimal timing and intensity of mobilization may differ according to the underlying neurological condition, and very early mobilization should not be assumed to be beneficial for all patients, particularly during the acute phase of stroke (47, 48). Accordingly, individualized assessment and multidisciplinary decision-making involving neurocritical care and rehabilitation professionals are particularly important in these patients.

Trauma represents another clinically important context in which EGDM should be considered on an individualized basis. Patients with severe or multiple trauma may experience prolonged immobilization and critical illness, both of which may contribute to neuromuscular weakness and functional decline. However, mobilization in this population may be additionally constrained by injury-specific factors, such as fracture stability, weight-bearing restrictions, spinal precautions, surgical wounds, and hemodynamic instability. Therefore, rather than applying a uniform mobilization protocol, EGDM for critically ill trauma patients should incorporate injury-specific safety restrictions into daily assessment, goal setting, and progression of mobilization (49, 50).

Patients with acute traumatic spinal cord injury (SCI), particularly those with high cervical injuries, represent another subgroup in whom respiratory impairment and prolonged ventilatory support may complicate rehabilitation. A recent systematic review and meta-analysis reported that early tracheostomy in patients with acute traumatic SCI was associated with shorter durations of mechanical ventilation and ICU and hospital lengths of stay, as well as lower rates of pneumonia and tracheostomy-related complications (51). Although these findings do not establish that early tracheostomy directly facilitates mobilization, reduced ventilatory dependence and shorter critical-care exposure may provide a more favorable clinical context for subsequent rehabilitation. Therefore, tracheostomy timing and respiratory stability may be relevant considerations when individualized mobilization goals are established for patients with SCI. Further studies are needed to evaluate EGDM specifically in neurocritical care populations and other high-risk subgroups, including critically ill trauma patients, and to determine diagnosis-specific timing, progression, and safety criteria.

These limitations also highlight several priorities for future research. More large-sample RCTs with rigorous methods and transparent reporting are needed, and future studies should provide detailed descriptions of the core components and contextual factors of the intervention to enable more informative subgroup analyses. Further research should also evaluate EGDM in specific neurocritical care and trauma populations and determine whether diagnosis-specific adaptations in timing, intensity, progression, and safety criteria are required.

## 5 Conclusion

Based on the available evidence, EGDM was associated with improvements in muscle strength and may also improve physical function in ICU patients. However, the certainty of the evidence varied across outcomes. Regarding the effect of EGDM on the incidence of ICU-AW, the available evidence from the highest-quality randomized controlled trials does not support a definitive conclusion. The observed effects on the duration of mechanical ventilation and ICU length of stay should also be interpreted cautiously because of the substantial between-study heterogeneity. In clinical practice, EGDM may be considered as an approach to support muscle strength and functional recovery in appropriately selected ICU patients. However, the current evidence is insufficient to establish EGDM as a standalone strategy for preventing ICU-AW. The present meta-analysis also does not provide direct evidence regarding the optimal timing of EGDM initiation or the effects of combining EGDM with sedation management or nutritional support. Further well-designed trials are needed to determine the optimal timing, dose, and implementation strategies for EGDM.

## Data Availability

The original contributions presented in the study are included in the article/Supplementary material, further inquiries can be directed to the corresponding authors.
