# Supplementary material for: Impact of early goal-directed mobilization on ICU-acquired weakness: a meta-analysis
Source: Front Neurol. 2026 Sep 30;17:1922493. doi: 10.3389/fneur.2026.1922493 (PMC13661485; doi:10.3389/fneur.2026.1922493)
Supplement: Supplementary file 1 [file Table_1.DOCX]

**Supplementary Table S1. Studies excluded after full-text assessment and reasons for exclusion**

| **No.** | **First author** | **Year** | **Study title** | **Reason for exclusion** |
| --- | --- | --- | --- | --- |
| 1 | Tian T | 2022 | Effect of an EGDM program on sleep quality and risk of ICU-acquired weakness in mechanically ventilated patients with respiratory failure | Did not meet the prespecified RCT design criterion: although described as an RCT, no random allocation procedure was reported in the full-text Methods; therefore, the study was excluded during eligibility screening before RoB 2 assessment. |
| 2 | Wang L | 2025 | Effect of a Structured Early Rehabilitation Program on Long-Term Functional Recovery, Quality of Life, and Survival in Patients With Severe Acute Pancreatitis: A 5-Year Randomized Controlled Trial | Trial registration record only; full-text study report unavailable at the time of the search |
| 3 | Hodgson CL | 2020 | Early Goal-Directed Mobilisation During Extracorporeal Membrane Oxygenation Improves Functional Outcome at Hospital Discharge, a Pilot Randomised Controlled Trial (The ECMO-PT Pilot Study) | Conference abstract only; full-text study report unavailable |
| 4 | Wu J | 2019 | Can in-reach multidisciplinary rehabilitation in the acute ward improve outcomes for critical care survivors? A pilot randomized controlled trial | Full text unavailable |
| 5 | Stevens RD | 2009 | A framework for diagnosing and classifying intensive care unit-acquired weakness | Full text unavailable |
| 6 | Song DW | 2025 | Effect of early goal-directed rehabilitation exercise combined with the ABCDEF bundle on the prevention of ICU-acquired weakness in mechanically ventilated ICU patients | Data errors |
| 7 | Chen D | 2021 | Effect of early goal-directed mobilization on the prevention of ICU-acquired weakness | Did not meet the prespecified RCT design criterion: although described as an RCT, no random allocation procedure was reported in the full-text Methods; therefore, the study was excluded during eligibility screening before RoB 2 assessment. |
| 8 | Wang D | 2024 | Application of an early goal-directed mobilization program in mechanically ventilated ICU patients with respiratory failure | Full text unavailable |
| 9 | Bian H | 2021 | Effect of an early goal-directed mobilization program on ICU-acquired weakness in mechanically ventilated ICU patients | Did not meet the prespecified RCT design criterion: group allocation was based on admission time rather than a random sequence; therefore, the study was excluded during eligibility screening before RoB 2 assessment. |
| 10 | Li K | 2024 | Effect of early rehabilitation exercise combined with the ABCDEF bundle on the prevention of ICU-acquired weakness in mechanically ventilated intensive care unit patients | Data errors |
| 11 | Wu JY | 2022 | Application of early mobilization combined with goal-directed sedation in mechanically ventilated patients with cancer | Did not meet the prespecified RCT design criterion: although described as an RCT, no random allocation procedure was reported in the full-text Methods; therefore, the study was excluded during eligibility screening before RoB 2 assessment. |
| 12 | Li Y | 2023 | Effect of early goal-directed mobilization on the prevention of deep vein thrombosis and ICU-acquired weakness in mechanically ventilated ICU patients | Did not meet the prespecified RCT design criterion: group allocation was based on admission time rather than a random sequence; therefore, the study was excluded during eligibility screening before RoB 2 assessment. |
| 13 | Zhou LH | 2023 | Application of a mechanical ventilation nursing management model based on early goal-directed mobilization in mechanically ventilated ICU patients | Did not meet the prespecified RCT design criterion: group allocation was based on admission time rather than a random sequence; therefore, the study was excluded during eligibility screening before RoB 2 assessment. |
| 14 | Li T | 2024 | Effect of goal-directed sequential early mobilization in patients with ICU-acquired weakness | Did not meet the prespecified RCT design criterion: group allocation was based on admission time rather than a random sequence; therefore, the study was excluded during eligibility screening before RoB 2 assessment. |

Abbreviations: EGDM, early goal-directed mobilization; ICU, intensive care unit; RoB 2.0, Risk of Bias 2 tool.
