# Supplementary material for: Impact of early goal-directed mobilization on ICU-acquired weakness: a meta-analysis
Source: Front Neurol. 2026 Sep 30;17:1922493. doi: 10.3389/fneur.2026.1922493 (PMC13661485; doi:10.3389/fneur.2026.1922493)
Supplement: Supplementary file 2 [file Table_2.DOCX]

**Supplementary Table S2. Detailed search strategies for each database**

| **Database** | **Complete search strategy** | **Controlled vocabulary** | **Fields** | **Limits/filters** | **Final search date** | **Records retrieved** |
| --- | --- | --- | --- | --- | --- | --- |
| CNKI | Topic (or Title/Keyword/Abstract) = ("early goal-directed" OR "goal-directed activity" OR "goal-directed rehabilitation" OR "goal-directed early activity" OR "structured early activity" OR "daily goal setting") AND Topic (or Title/Keyword/Abstract) = ("ICU" OR "intensive care" OR "intensive care unit" OR "mechanical ventilation") AND Topic (or Title/Keyword/Abstract) = ("ICU-acquired weakness" OR "ICU-AW" OR "muscle weakness") AND Title/Keyword/Abstract = ("randomized" OR "RCT") | None | Topic; Title/Keyword/Abstract | No additional database filters | 2025-11-30 | 6 |
| Wanfang Data | (Title or Keyword = ("early goal-directed" OR "goal-directed activity" OR "goal-directed rehabilitation" OR "structured early activity") OR Abstract = ("daily goal" OR "goal setting")) AND Title or Keyword = ("ICU" OR "intensive care" OR "intensive care unit" OR "mechanical ventilation") AND Title or Keyword = ("ICU-acquired weakness" OR "ICU-AW" OR "muscle weakness") AND Abstract = ("randomized" OR "RCT") | None | Title/Keyword; Abstract | No additional database filters | 2025-11-30 | 8 |
| VIP | M = ("early goal-directed" OR "goal-directed activity" OR "goal-directed rehabilitation" OR "structured early activity" OR "daily goal setting") AND M = ("ICU" OR "intensive care" OR "intensive care unit" OR "mechanical ventilation") AND M = ("ICU-acquired weakness" OR "ICU-AW" OR "muscle weakness") AND R = ("randomized" OR "RCT") | None | M field; R field | No additional database filters | 2025-11-30 | 24 |
| PubMed | ((("goal-directed"[Title/Abstract] OR "goal oriented"[Title/Abstract] OR "target-directed"[Title/Abstract]) AND ("mobilization"[Title/Abstract] OR "mobilisation"[Title/Abstract] OR "activity"[Title/Abstract] OR "rehabilitation"[Title/Abstract] OR "exercise"[Title/Abstract])) OR ("Early Ambulation"[Mesh] OR "Rehabilitation"[Mesh])) AND ("Intensive Care Units"[Mesh] OR "Critical Care"[Mesh] OR "Respiration, Artificial"[Mesh] OR "intensive care unit"[Title/Abstract] OR "ICU"[Title/Abstract] OR "critical care"[Title/Abstract] OR "mechanical ventilation"[Title/Abstract]) AND ("randomized controlled trial"[Publication Type] OR "randomized"[Title/Abstract] OR "randomised"[Title/Abstract] OR "RCT"[Title/Abstract]) | MeSH: Early Ambulation; Rehabilitation; Intensive Care Units; Critical Care; Respiration, Artificial | Title/Abstract; MeSH; Publication Type | No additional database filters | 2025-11-30 | 1,091 |
| Web of Science | TS=("goal-directed" OR "goal oriented" OR "target-directed") AND TS=("intensive care unit" OR "ICU" OR "critical care" OR "mechanical ventilation") AND TS=("mobilization" OR "mobilisation" OR "activity" OR "rehabilitation" OR "exercise") AND AB=("randomized controlled trial" OR "randomised controlled trial" OR "RCT") | None | TS (Topic); AB (Abstract) | No additional database filters | 2025-11-30 | 5 |
| Cochrane Library | "goal-directed" OR "goal oriented" OR "target-directed" in Title Abstract Keyword AND "mobilization" OR "mobilisation" OR "activity" OR "rehabilitation" OR "exercise" in Title Abstract Keyword AND "intensive care unit" OR "ICU" OR "critical care" OR "mechanical ventilation" in Title Abstract Keyword AND "randomized" OR "randomised" OR "RCT" NOT protocol in Title Abstract Keyword - (Word variations have been searched) | None in the recorded search strategy | Title; Abstract; Keyword | Protocols excluded in the search expression; word variations searched | 2025-11-30 | 53 |
| Embase | ('early ambulation'/exp OR 'early ambulation' OR 'rehabilitation'/exp OR 'rehabilitation' OR ((early NEAR/3 (mobili*ation OR rehabilitation OR exercise OR activity)):ti,ab,kw)) AND ('goal directed':ti,ab,kw OR 'goal-directed':ti,ab,kw OR 'goal oriented':ti,ab,kw OR 'goal-oriented':ti,ab,kw OR 'target-directed':ti,ab,kw OR 'daily goal':ti,ab,kw OR 'goal setting':ti,ab,kw OR 'protocolized':ti,ab,kw OR 'structured':ti,ab,kw) AND ('intensive care unit'/exp OR 'intensive care unit' OR 'critical care'/exp OR 'critical care' OR 'mechanical ventilation'/exp OR 'mechanical ventilation' OR icu:ti,ab,kw OR 'intensive care':ti,ab,kw OR 'critical care':ti,ab,kw OR 'mechanical ventilation':ti,ab,kw) AND ('intensive care unit acquired weakness'/exp OR 'intensive care unit acquired weakness' OR 'muscle weakness'/exp OR 'muscle weakness' OR 'polyneuropathy'/exp OR 'polyneuropathy' OR 'icu acquired weakness':ti,ab,kw OR 'icu-aw':ti,ab,kw OR 'critical illness polyneuropathy':ti,ab,kw OR 'critical illness myopathy':ti,ab,kw OR 'muscle weakness':ti,ab,kw) AND ('randomized controlled trial'/exp OR 'randomized controlled trial' OR random*:ti,ab,kw) | Emtree (/exp): early ambulation; rehabilitation; intensive care unit; critical care; mechanical ventilation; intensive care unit acquired weakness; muscle weakness; polyneuropathy; randomized controlled trial | ti, ab, kw; Emtree explosion (/exp) | No additional database filters | 2025-11-30 | 76 |
| **Total records retrieved** | | | | | | **1,263** |

Note: Search strategies for CNKI, Wanfang Data, and VIP are presented as English translations of the original Chinese-language search strategies; the original search logic and field restrictions were retained. The final search date was November 30, 2025, for all databases. “No additional database filters” indicates that no filters beyond those explicitly embedded in the recorded search expression were applied.
