# Supplementary material for: Impact of early goal-directed mobilization on ICU-acquired weakness: a meta-analysis
Source: Front Neurol. 2026 Sep 30;17:1922493. doi: 10.3389/fneur.2026.1922493 (PMC13661485; doi:10.3389/fneur.2026.1922493)
Supplement: Supplementary file 3 [file Table_3.DOCX]

**Supplementary Table S3. Study-level definition and ascertainment of ICU-acquired weakness**

| Study | ICU-AW definition | Assessment instrument | Diagnostic threshold | Assessment timing | Assessor / blinding | Additional ascertainment details |
| --- | --- | --- | --- | --- | --- | --- |
| Hodgson^[21]^ | ICU-AW was defined as an MRC-SS <48. | Medical Research Council Sum Score (MRC-SS) | <48/60 | At ICU discharge | ICU discharge strength/function assessment was blinded; the trial was assessor-blinded. | Participants had to be able to follow simple verbal commands; ICU-AW incidence was based on the discharge MRC-SS. |
| Lin^[22]^ | ICU-AW was diagnosed when the MRC score was <48. | Medical Research Council (MRC) scale assessing six bilateral muscle groups | <48/60 | At transfer from the ICU and at hospital discharge; the meta-analysis used the ICU-discharge time point (T1). | In-hospital data were collected by a nurse unaware of group assignment. | The study reported ICU-AW at both ICU discharge (T1: 8/39 vs 16/38) and hospital discharge (T2: 3/39 vs 11/38). |
| Schaller^[15]^ | Clinically significant muscle weakness was defined as an MRC sum score <48; this threshold was used for ICU-AW ascertainment in the meta-analysis. | Medical Research Council (MRC) sum score | <48/60 | Once weekly while the patient was in the SICU | Administered by trained study staff; blinding of the MRC assessor was not explicitly reported. | The original article describes the threshold as 'clinically significant muscle weakness' rather than explicitly labeling it ICU-AW. |
| Gu^[26]^ | ICU-AW was diagnosed when the MRC score was <48. | Medical Research Council (MRC) scale assessing six muscle groups | <48/60 | After the intervention; the exact assessment time point was not reported. | NR | Participants were required to be conscious and able to communicate and cooperate with assessment/intervention. |
| He^[25]^ | ICU-AW was described as unexplained generalized limb weakness acquired during ICU stay; MRC score <48 indicated ICU-AW. | Medical Research Council (MRC) scale assessing six bilateral muscle groups | <48/60 | After the intervention; the exact assessment time point was not reported. | NR | The study reported pre-/post-intervention MRC scores and post-intervention ICU-AW incidence. |

Note: ICU-AW, intensive care unit-acquired weakness; MRC, Medical Research Council; MRC-SS, Medical Research Council Sum Score; SICU, surgical intensive care unit; NR, not reported. Where the original report did not specify the assessor or exact timing, this was recorded as NR rather than inferred.
